# Supplementary material for: Ultrasound-responsive low-dose doxorubicin liposomes trigger mitochondrial DNA release and activate cGAS-STING-mediated antitumour immunity
Source: Nat Commun. 2023 Jun 30;14:3877. doi: 10.1038/s41467-023-39607-x (PMC10313815; doi:10.1038/s41467-023-39607-x)
Supplement: Supplementary file 1 — Supplementary Information [file 41467_2023_39607_MOESM1_ESM.pdf]

Supplementary Information

Ultrasound-responsive low-dose doxorubicin liposomes trigger mitochondrial DNA release and activate cGAS-STING mediated antitumour immunity

Chaoyu Wang[1,2], Ruoshi Zhang[1,2], Jia He[1,2], Lvshan Yu[1,2], Xinyan Li[1,2], Junxia Zhang[2,3,4], Sai Li[2,3,4], Conggang Zhang[1,2], Jonathan C. Kagan[5], Jeffrey M. Karp[6,7,8,9], Rui Kuai[1,2,*]

[1]School of Pharmaceutical Sciences, Tsinghua University, Beijing 100084, China.

[2]Tsinghua-Peking Center for Life Sciences, Beijing 100084, China.

[3]School of Life Sciences, Tsinghua University, Beijing 100084, China.

[4]Frontier Research Center for Biological Structure & State Key Laboratory of Membrane Biology, Beijing 100084, China.

[5]Division of Gastroenterology, Boston Children's Hospital and Harvard Medical School, Boston, MA, USA.

[6]Department of Anesthesiology, Perioperative, and Pain Medicine, Brigham and Women's Hospital, Harvard Medical School, Boston, MA, USA.

[7]Harvard-MIT Program in Health Sciences and Technology, MIT, Cambridge, MA, USA.

[8]Harvard Stem Cell Institute, Harvard University, Cambridge, MA, USA.

[9]Broad Institute of MIT and Harvard, Cambridge, MA, USA.

*Corresponding author. Email:ruikuai@tsinghua.edu.cn

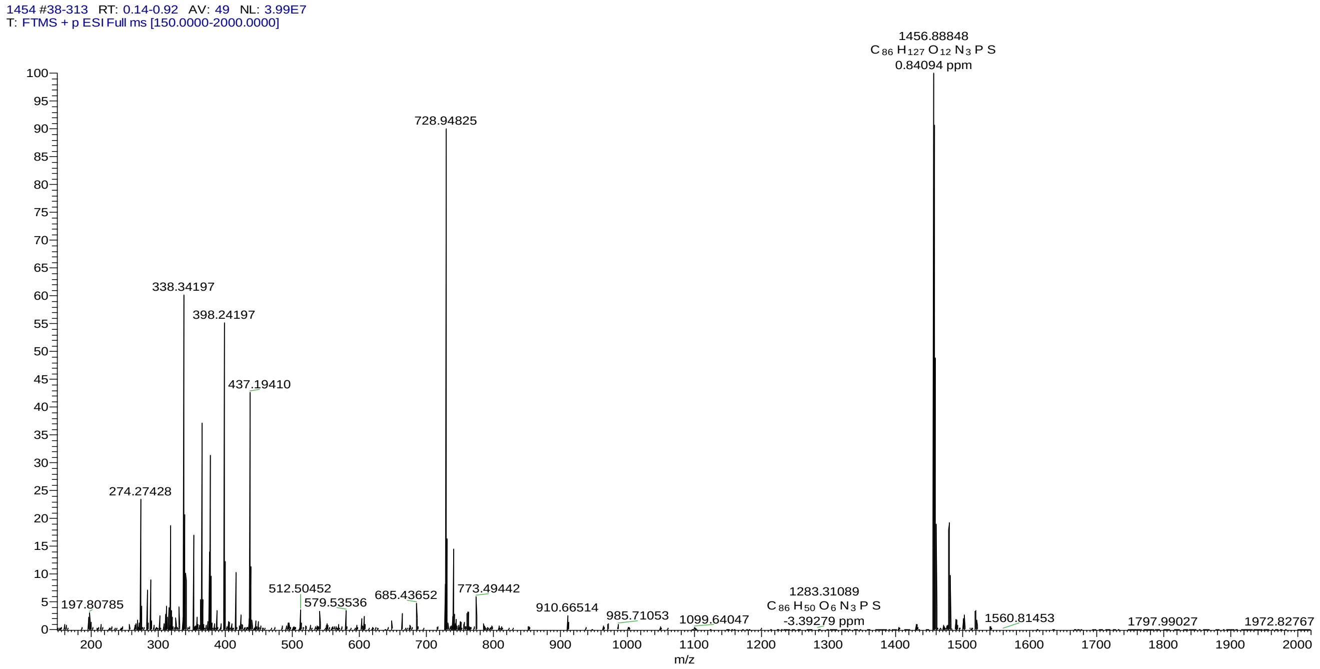

Supplementary Fig. 1. Mass spectrum of DOPE-ICG.

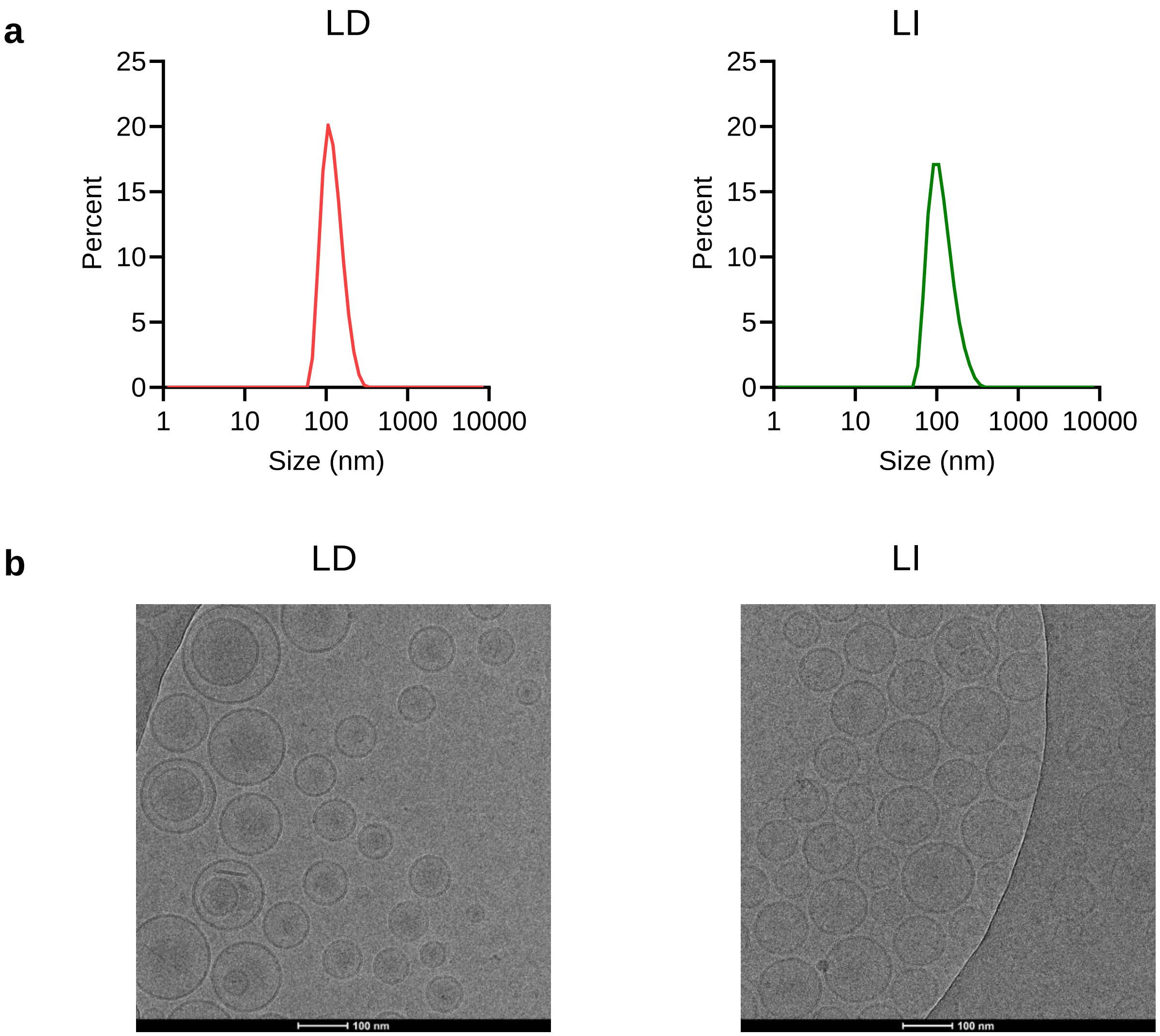

Supplementary Fig. 2. Characterization of LD and LI. (**a**) Size distribution of liposomal DOX (LD) and liposomal ICG (LI) . (**b**) Cryo-electron microscopy (cryo-EM) of LD and LI. Source data are provided as a Source Data file. The data are representative of two independent experiments.

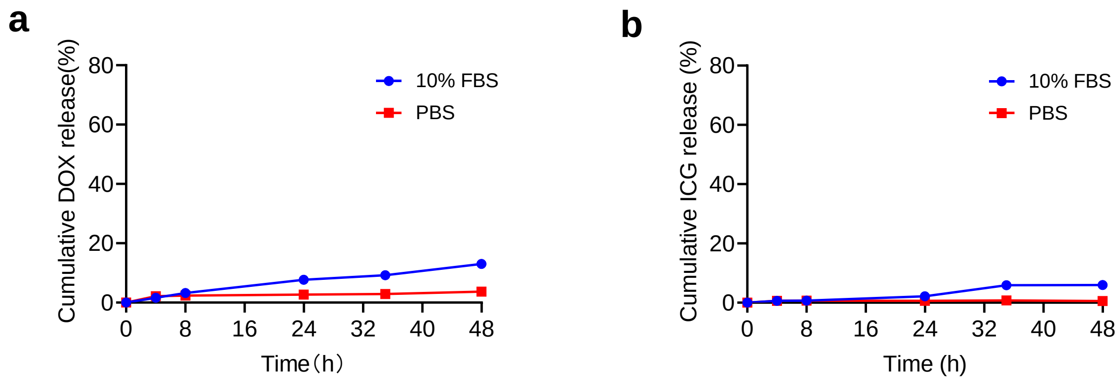

Supplementary Fig. 3. Stability of LID. (a) The release of DOX in PBS or 10% FBS containing PBS at 37 ℃. (b) The release of ICG in PBS or 10% FBS containing PBS at 37 ℃. Data represent mean ± SEM (n = 3 experimental replicates per group). Source data are provided as a Source Data file.

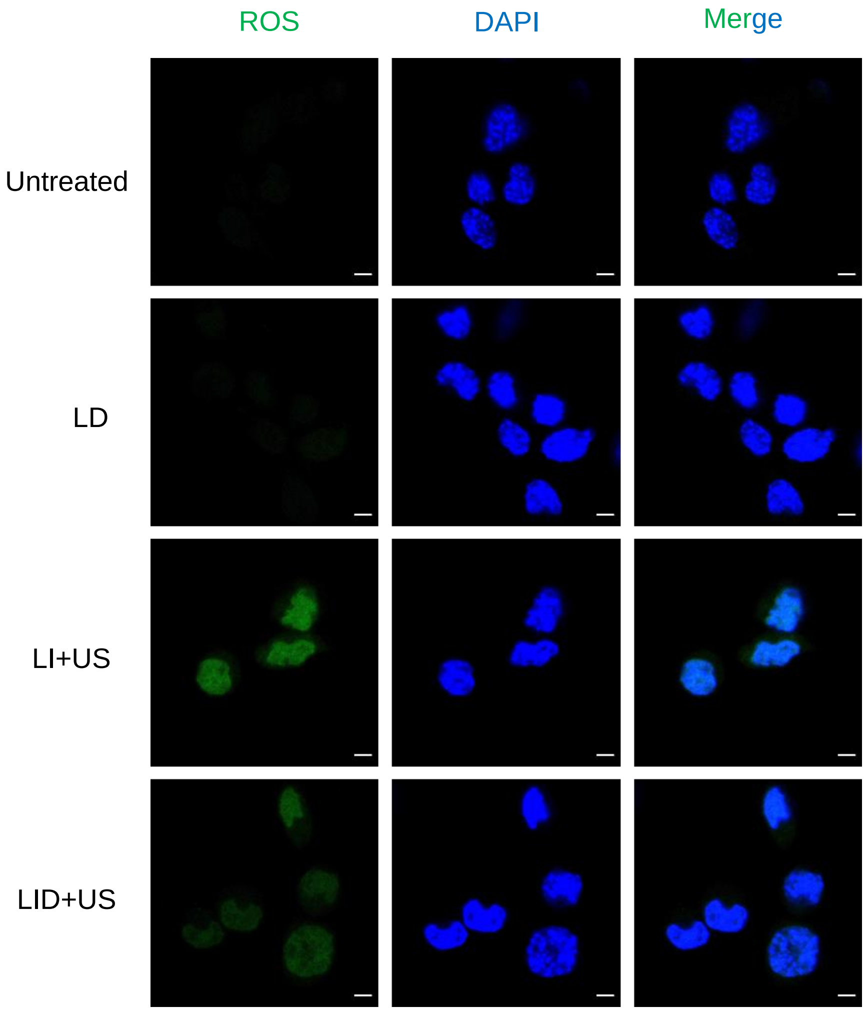

Supplementary Fig. 4. LID-induced ROS generation in MC38 cells. ROS generation in MC38 induced by indicated formulations with or without ultrasound (US). Scale bar = 10 μm. The data are representative of two independent experiments.

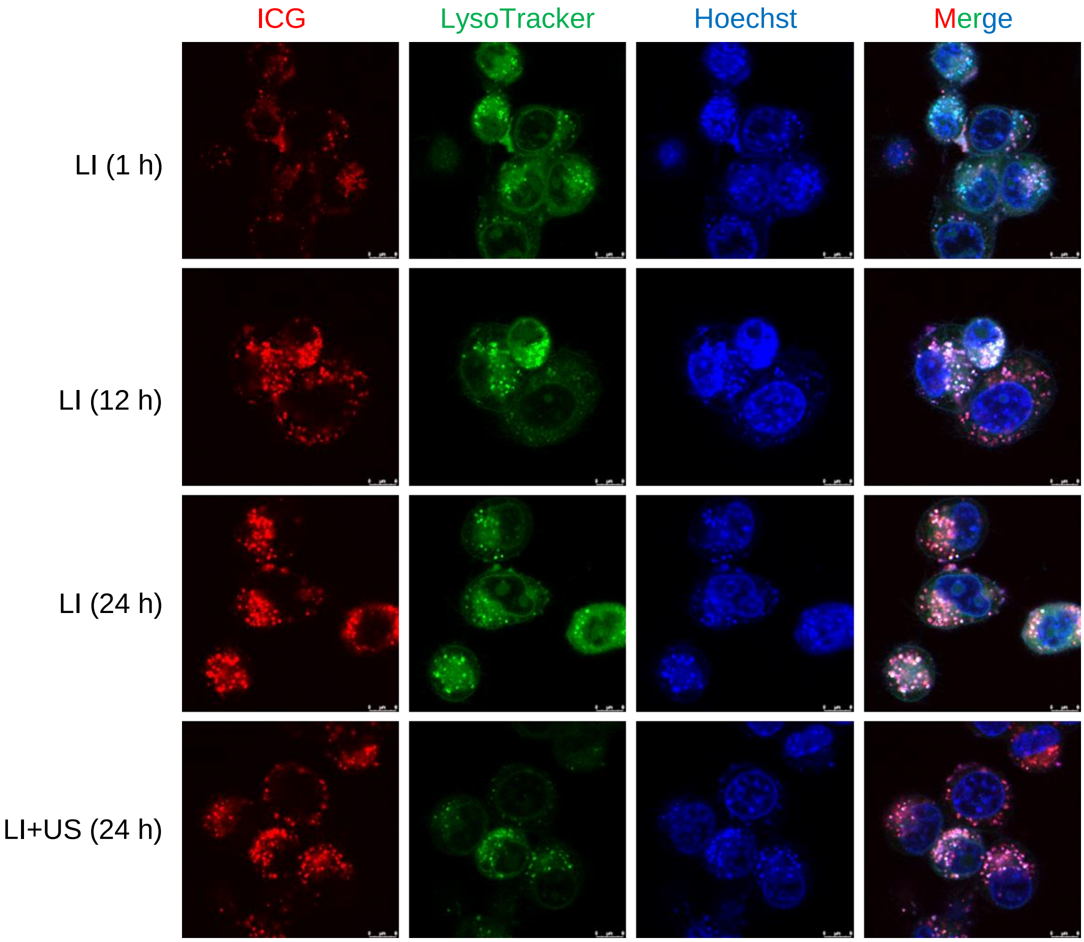

Supplementary Fig. 5. Intracellular delivery profile of ICG. Confocal microscopy of LI (or LI+US) in CT26 tumor cells at different time points. Shown are the signal from ICG (red) and lysosomes (green). The nuclei were stained by Hoechst. Scale bar = 10 μm. The data are representative of two independent experiments.

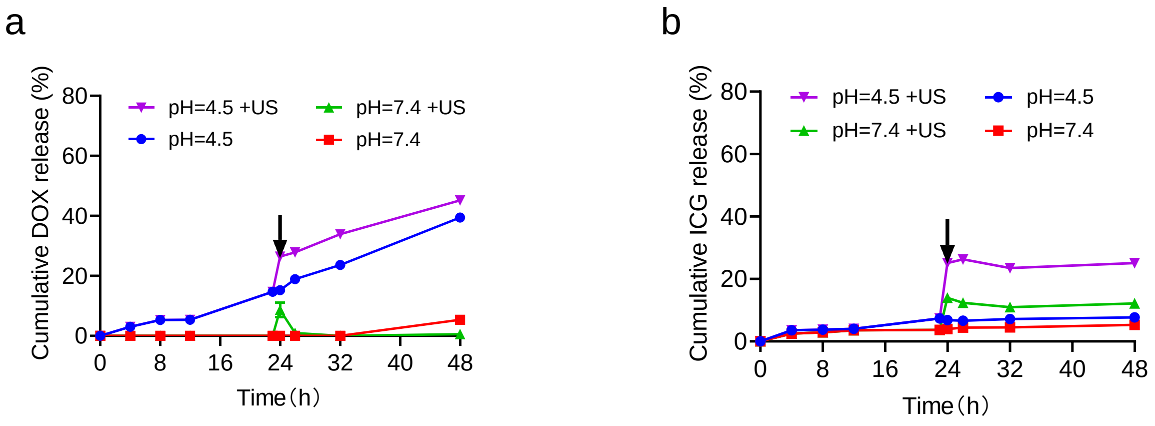

Supplementary Fig. 6. Effect of pH and ultrasound on DOX and ICG release from LID. (a) DOX release from LID under indicated conditions. (b) ICG release from LID under indicated conditions. Data represent mean ± SEM (n = 3 experimental replicates per group). Source data are provided as a Source Data file.

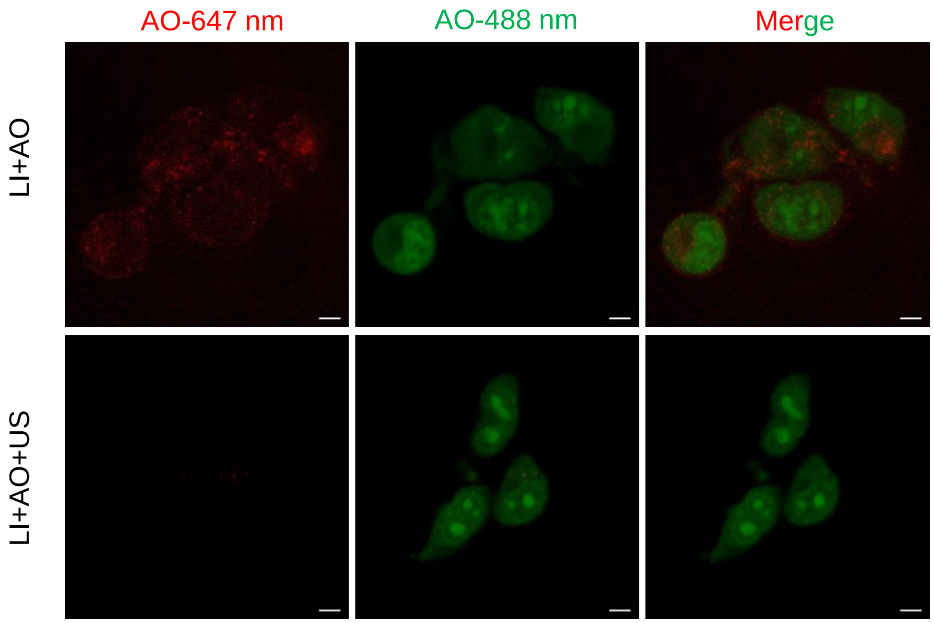

Supplementary Fig. 7. Analysis of endosomal membrane permeability. 50,000 MC38 tumor cells were treated with LI for 24 h. Ultrasound (2 W/cm^2, 50% duty cycle, 5 min) was applied to the selected group (LI+US). Acridine Orange (AO, 5 μM) was added immediately after ultrasound treatment and incubated for 30 min at 37℃ before confocal microscopy. Scale bar = 10 μm. The data are representative of two independent experiments.

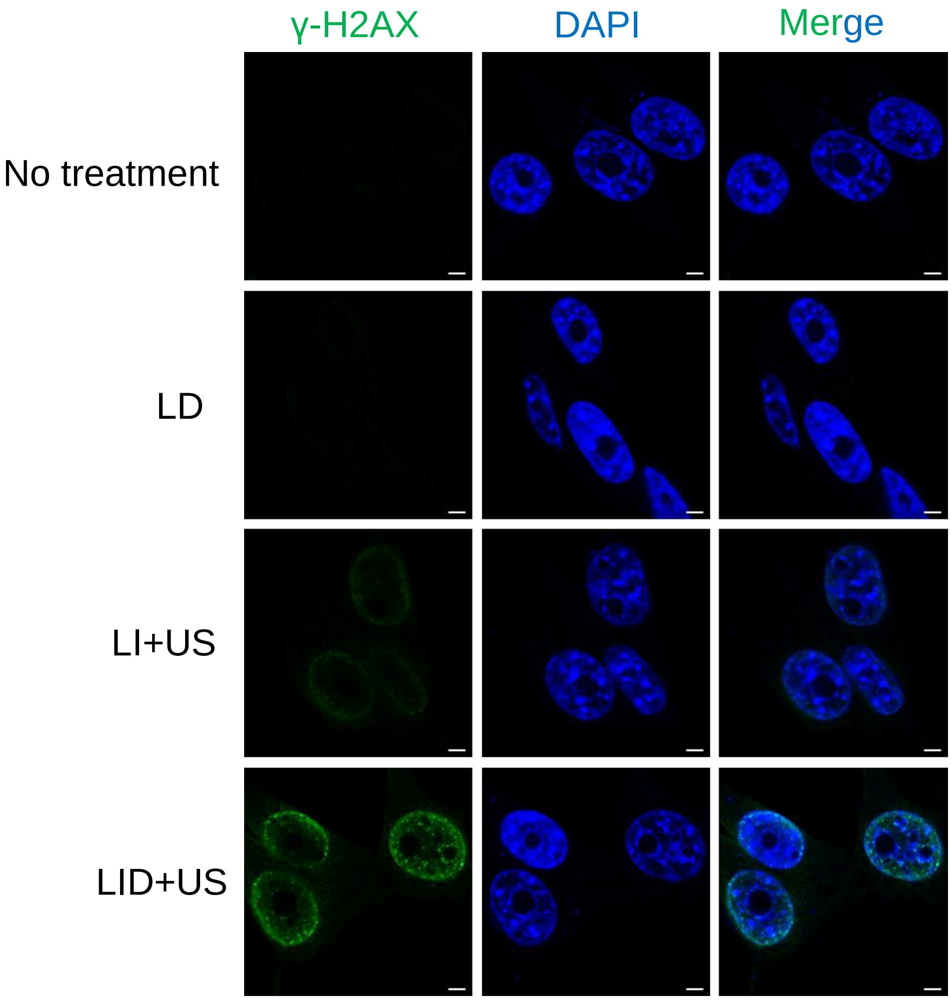

Supplementary Fig. 8. Analysis of γ-H2AX in the nuclei of CT26 cells. Confocal microscopy for γ-H2AX after CT26 tumor cells were treated with indicated formulations. Scale bar = 10 μm. The data are representative of two independent experiments.

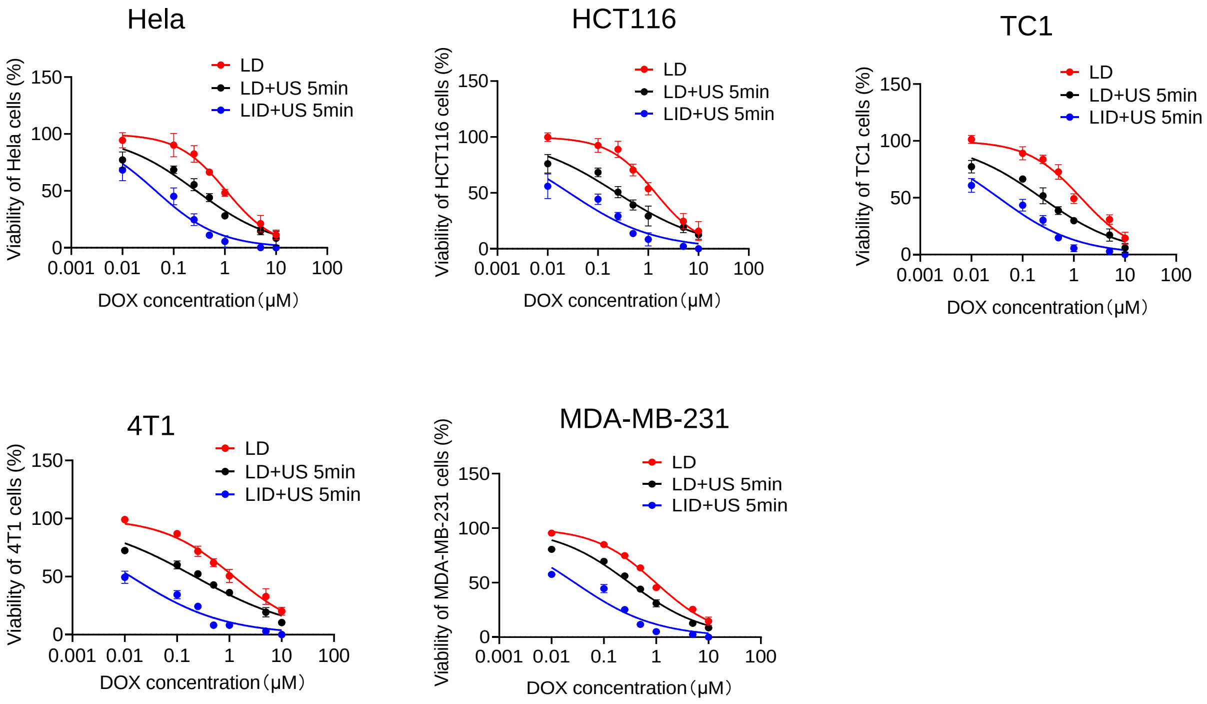

Supplementary Fig. 9. Cytotoxic effect of indicated formulations on Hela, HCT1116, TC1 cells, 4T1, and MDA-MB-231 cells. Data represent mean ± SEM (n = 3 experimental replicates per group). Source data are provided as a Source Data file.

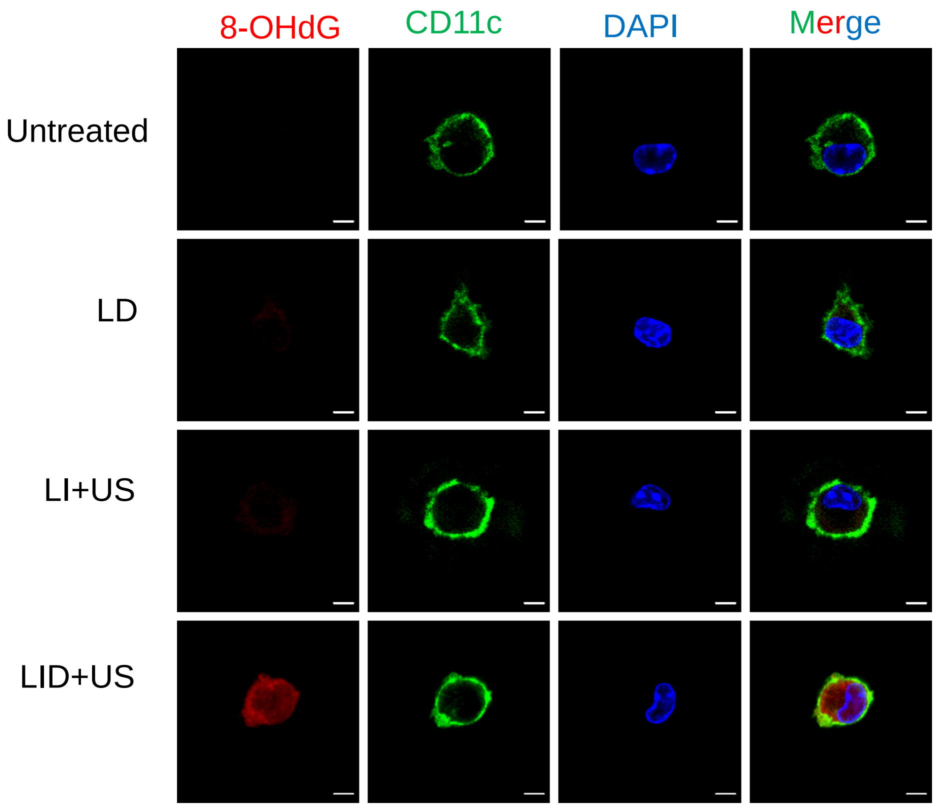

Supplementary Fig. 10. Transfer of oxidized tumor DNA to dendritic cells. CT26 cells were treated with indicated formulations for 24 h. Ultrasound (2 W/cm^2, 50% duty cycle, 5 min) was applied to selected groups. After 24 h, BMDCs were added and co-cultured for another 24 h, followed by staining with anti-8OHD and CD11c antibodies before confocal microscopy. Scale bars, 5 μm. The data are representative of two independent experiments.

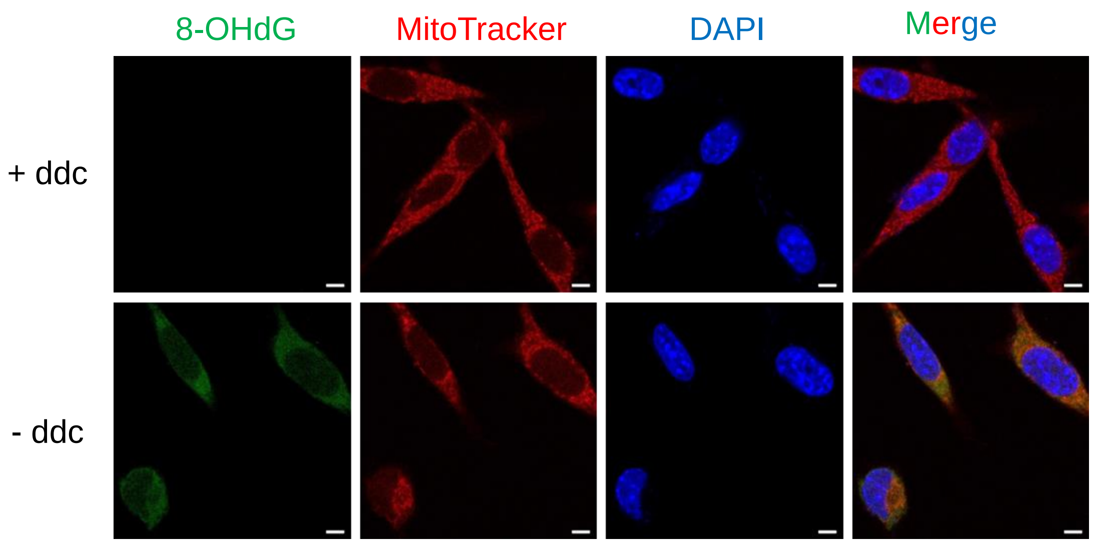

Supplementary Fig. 11. Mitochondrial DNA depletion by ddc. CT26 cells were treated with or without 150 μM ddc for 6 days. These CT26 cells were then treated with the same LID for 24 h, followed by US treatment (2 W/cm^2, 50%, 1 MHz, 5 min). After another 24 h, mitochondrial DNA was stained by the anti 8-OHdG antibody and mitochondria were stained by the mitoTracker. Scale bar = 10 μm. The data are representative of two independent experiments.

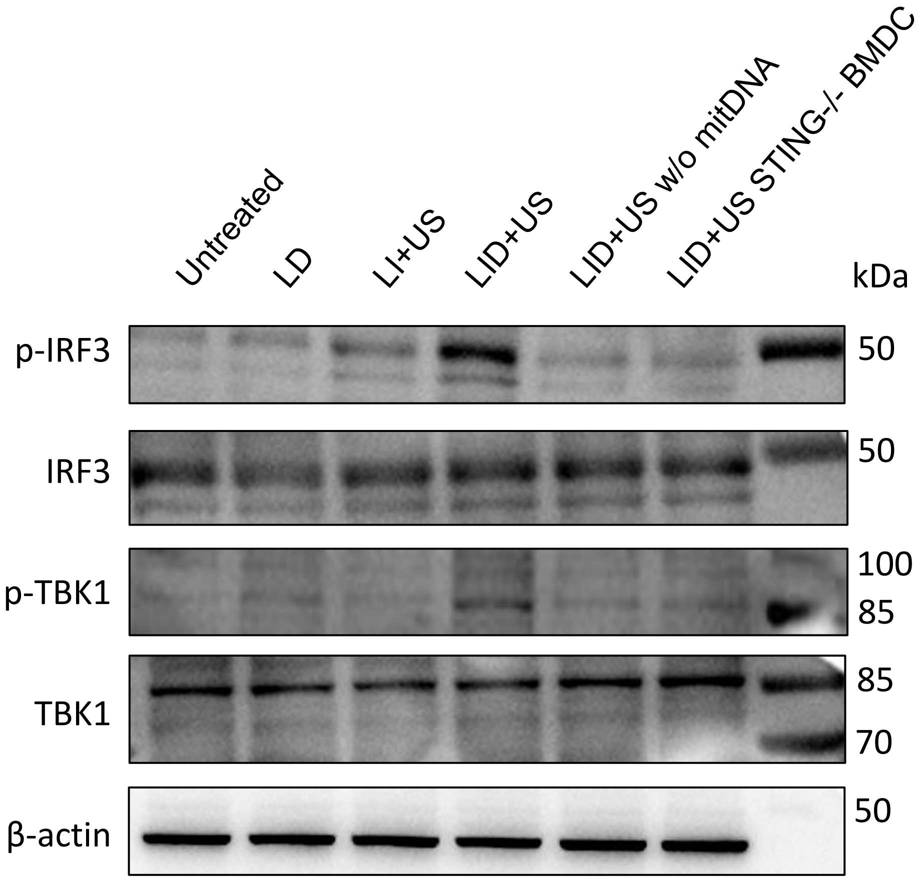

Supplementary Fig. 12. Activation of STING pathway in BMDC after treatment with different formulations. MC38 tumor cells or MC38 tumor cells with mitochondrial DNA depleted by ddc were treated with indicated formulations. Ultrasound (2 W/cm^2, 50% duty cycle, 5 min) was applied to selected groups. After 24 h, WT BMDC or STING-/- BMDC were added to tumor cells and cocultured for another 24 h, followed by western blot analysis of indicated markers. The data are representative of two independent experiments.

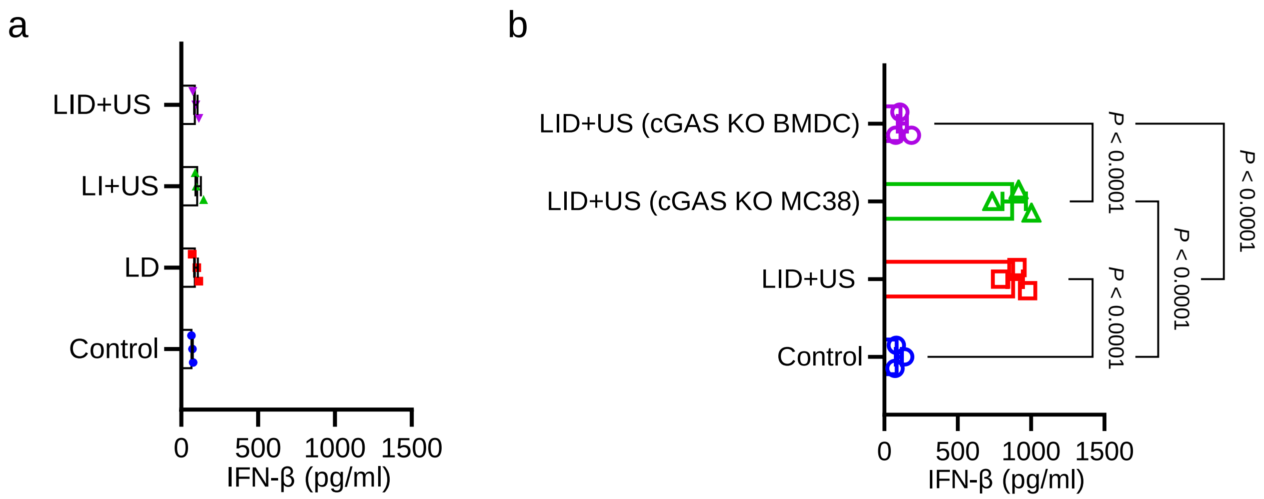

Supplementary Fig. 13. Transfer of DNA to BMDC is critical for IFNβ secretion. (a) MC38 tumor cells were treated with indicated formulations for 24 h. Ultrasound (2 W/cm^2, 50% duty cycle, 5 min) was applied to selected groups. After 24 h, IFNβ in the supernatant was measured using the ELISA kit (n = 3 experimental replicates per group). (b) MC38 or cGAS KO MC38 tumor cells were treated with indicated formulations for 24 h. Ultrasound (2 W/cm2, 50% duty cycle, 5 min) was applied to selected groups. After 24 h, BMDCs or cGAS KO BMDC were added and co-cultured for another 24 h, followed by measuring IFNβ using the ELISA kit (n = 3 experimental replicates per group). Data represent mean ± SEM (**a-b**). Data were analyzed by one-way analysis of variance (ANOVA) with Tukey's multiple comparisons post test. Source data are provided as a Source Data file.

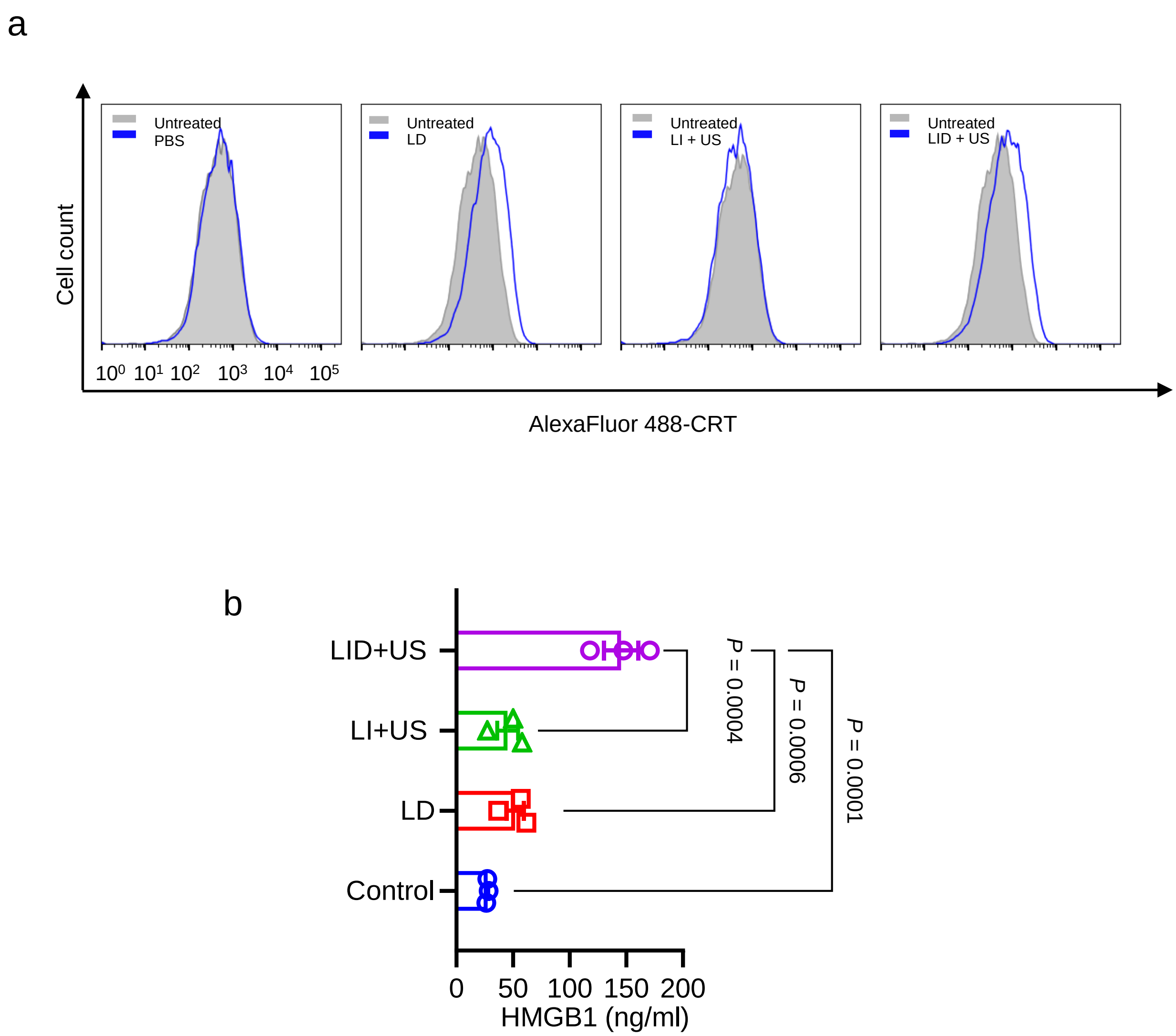

Supplementary Fig. 14. CRT and HMGB1 levels after treating MC38 cells with different formulations. 200,000 MC38 tumor cells were treated with indicated formulations for 24 h. Ultrasound (2 W/cm^2, 50% duty cycle, 5 min) was applied to selected groups, followed by measuring CRT levels (**a**) using flow cytometry or HMGB1 (**b**) using the ELISA kit following the manufacturer's instructions. Data are representative of three independent experiments (**a**) or represent mean ± SEM (n = 3 experimental replicates per group) (**b**). Data were analyzed by one-way analysis of variance (ANOVA) with Tukey's multiple comparisons post test. Source data are provided as a Source Data file.

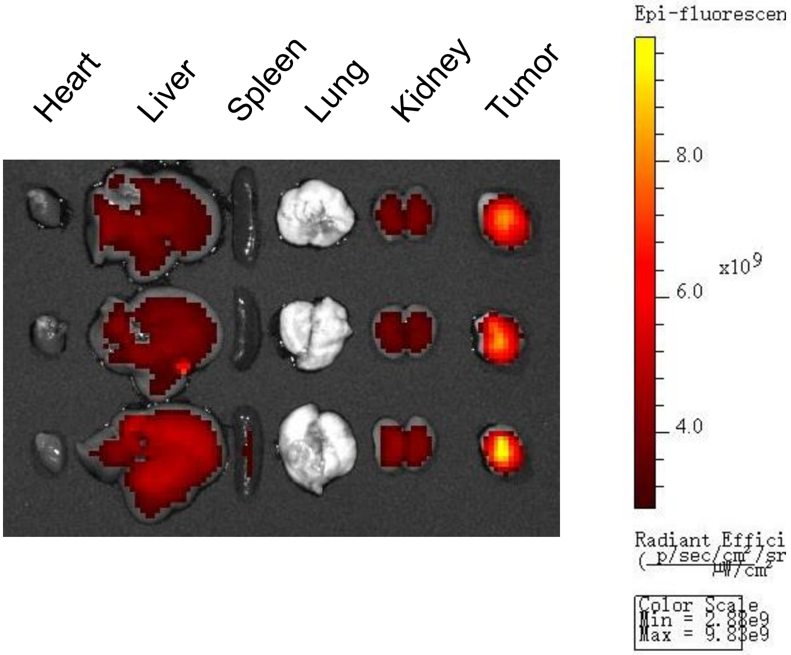

Supplementary Fig. 15. Biodistribution of LID in MC38 tumor-bearing mice. MC38 tumor-bearing mice were intravenously injected with LID and the animals were euthanized and major organs were harvested for the IVIS optical imaging system 24 h post injection. Data are representative of two independent experiments.

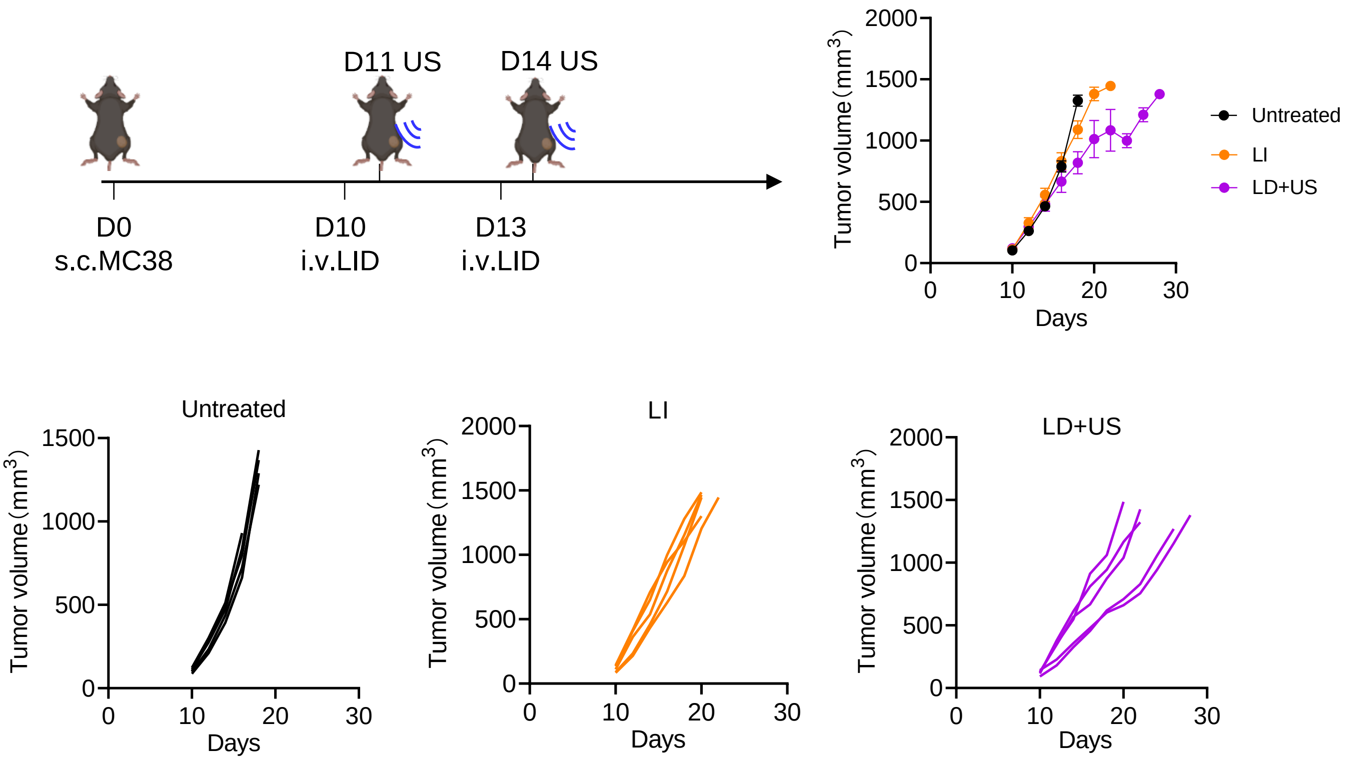

Supplementary Fig. 16. Therapeutic effect on MC38 tumors. C57BL/6 mice were subcutaneously injected with 500,000 MC38 cells on day 0. On days 10 and 13, tumor-bearing mice were i.v. injected with LI or LD (DOX 0.5 mg/kg, ICG 4 mg/kg) or control formulations. On days 11 and 14, ultrasound（2 W/cm^2, 50%, 1 MHz, 5 min）was performed for selected groups. Shown are average and individual tumor growth curves for MC38 tumor-bearing mice treated with indicated formulations (n = 5 mice per group). Data represent mean ± SEM. Source data are provided as a Source Data file.

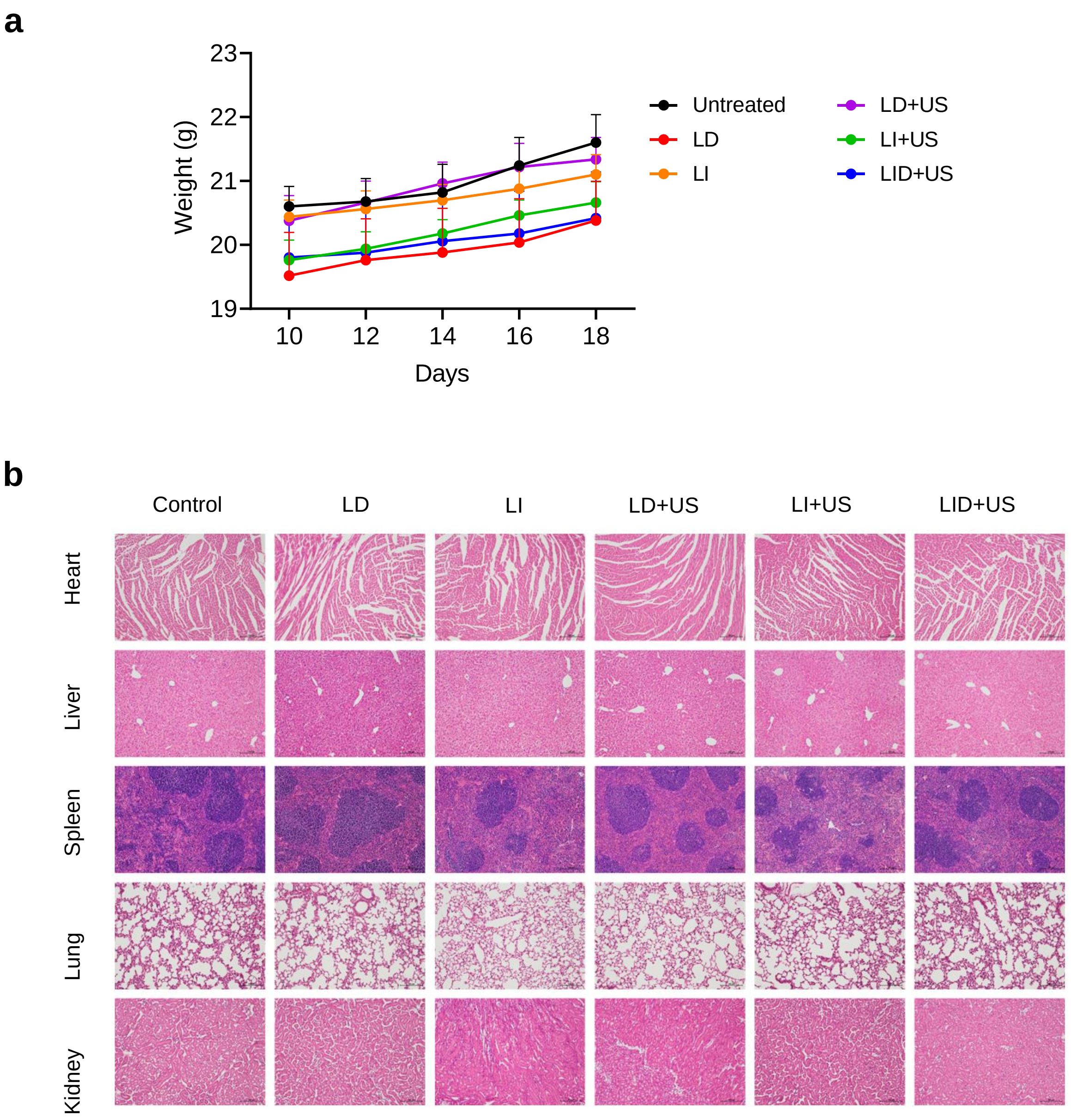

Supplementary Fig. 17. Safety profile of LID. C57BL/6 mice were subcutaneously injected with 500,000 MC38 cells on day 0. On days 10 and 13, tumor-bearing mice were i.v. injected indicated formulations (DOX 0.5 mg/kg, ICG 4 mg/kg). On days 11 and 14, ultrasound（2 W/cm^2, 50%, 1 MHz, 5 min) was performed for selected groups. Shown are (a) body weights for indicated groups (n = 5 mice per group). Data represent mean ± SEM. (b) hematoxylin and eosin (H&E) staining of the major organs harvested on day 20 from tumor-bearing mice treated with indicated formulations. Data are representative of two independent experiments. Source data are provided as a Source Data file.

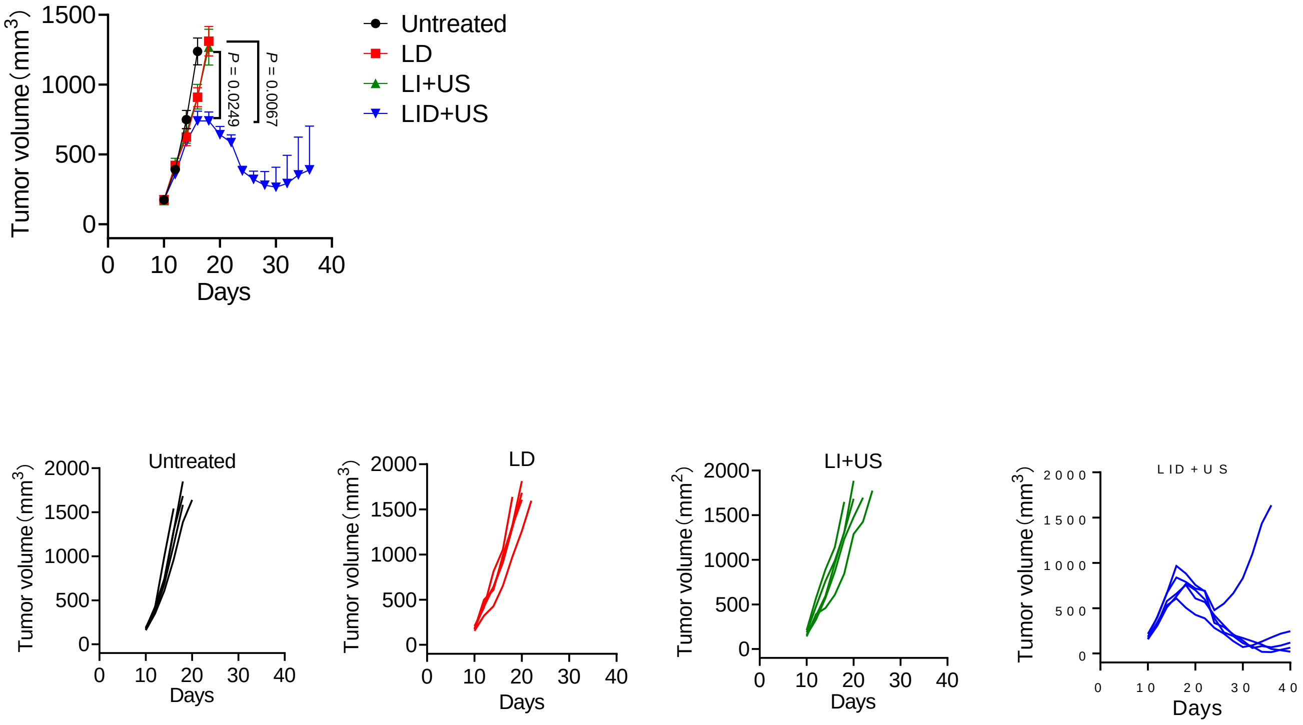

Supplementary Fig. 18. Therapeutic effect on CT26 tumors. Balb/c mice were subcutaneously injected with 200,000 CT26 cells on day 0. On days 10, 13, and 16, tumor-bearing mice were i.v. injected with LID (DOX 0.5 mg/kg, ICG 4 mg/kg) or control formulations. On days 11, 14, and 17, ultrasound (2 W/cm^2, 50%, 1 MHz, 5 min was performed for selected groups. Shown are average and individual tumor growth curves for CT26 tumor-bearing mice treated with indicated formulations. Data were analyzed by two-way ANOVA with Tukey's multiple comparisons post test. Data represent mean ± SEM (n = 5 mice per group). Source data are provided as a Source Data file.

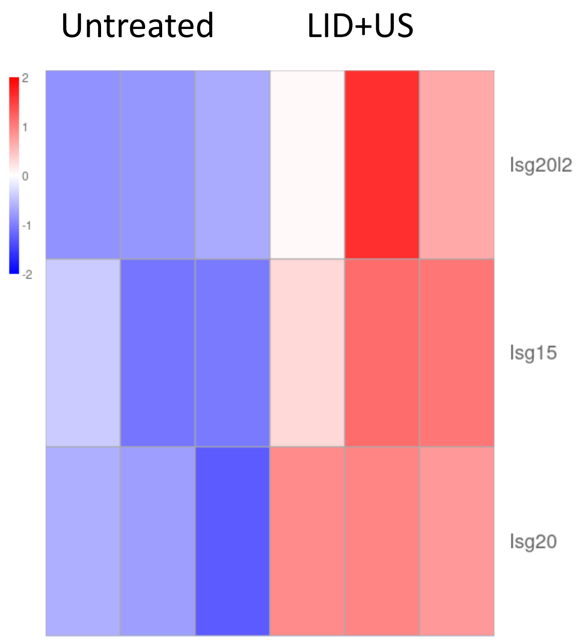

Supplementary Fig. 19. ISG levels in the tumor tissues from untreated animals or LID+US treated animals. C57BL/6 mice were subcutaneously injected with 500,000 MC38 cells on day 0. On days 10 and 13, tumor-bearing mice were i.v. injected with LID (DOX 0.5 mg/kg, ICG 4 mg/kg) or untreated. On days 11 and 14, ultrasound (2 W/cm^2, 50%, 1 MHz, 5 min) was performed. Shown are ISG levels in the tumor for indicated groups (n = 3 mice per group).

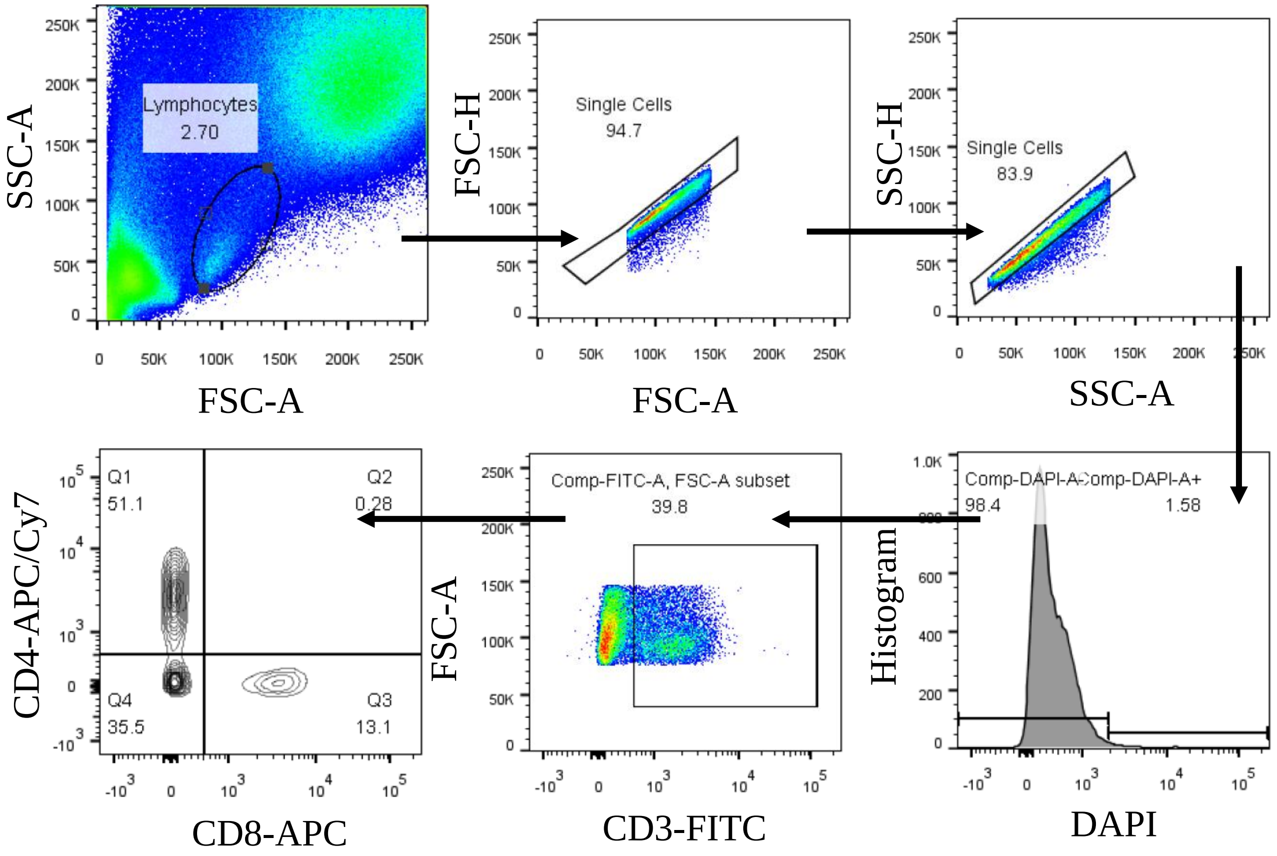

Supplementary Fig. 20. Flow cytometry gating strategy for analysis of CD8+ and CD4+ T cells in the tumor.

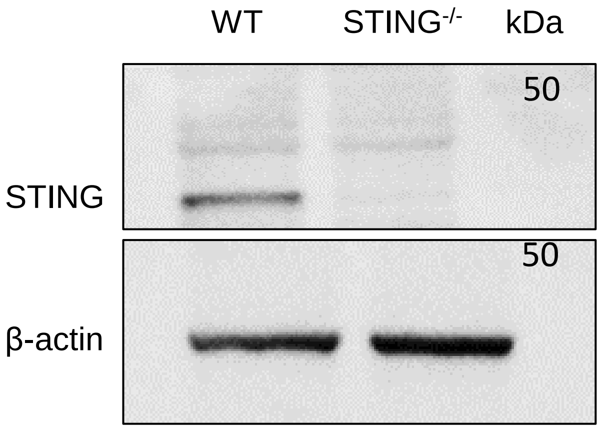

Supplementary Fig. 21. Western blot for WT MC38 tumor cells and STING$^{-/-}$ MC38 tumor cells. The data are representative of two independent experiments.

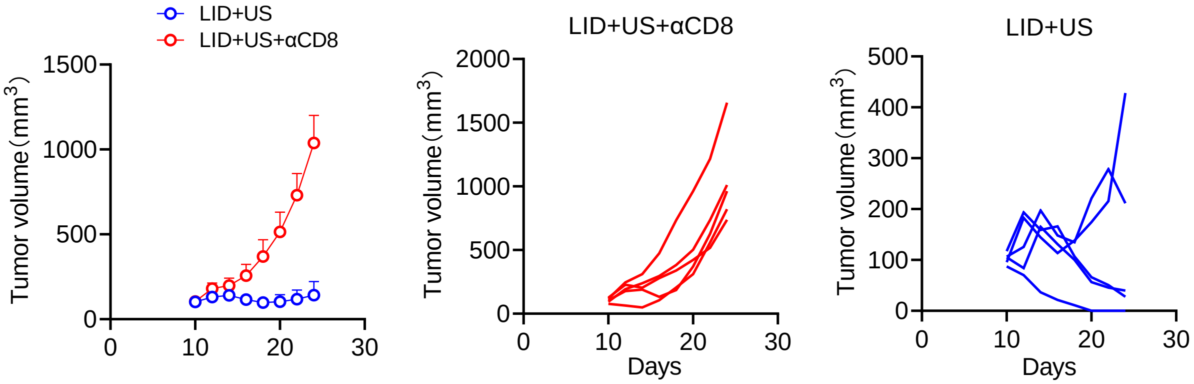

Supplementary Fig. 22. Effect of CD8+ T cell depletion on the therapeutic efficacy of LID+US. C57BL/6 mice were subcutaneously injected with 500,000 MC38 cells on day 0. On days 10 and 13, tumor-bearing mice were i.v. injected with LID. On days 11 and 14, ultrasound (2 W/cm^2, 50%, 1 MHz, 5 min) was applied to the tumor region. On days -2, 0, 2, 6, 13, and 20 tumor-bearing mice were i.p. injected with αCD8 (200 μg) for the indicated group. Shown are average and individual tumor growth curves for MC38 tumor-bearing mice treated with indicated formulations (n = 5 mice per group). Data represent mean ± SEM. Source data are provided as a Source Data file.

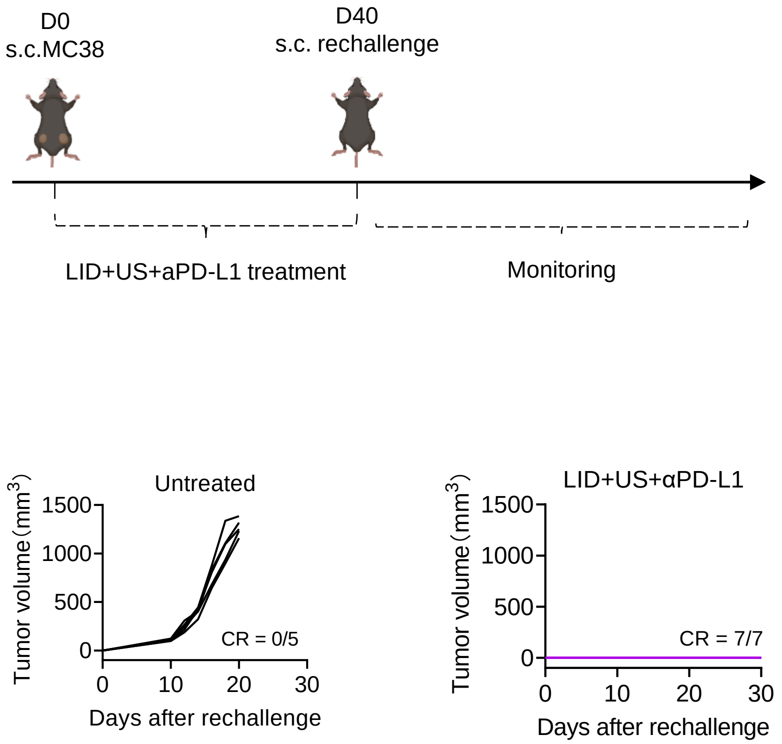

Supplementary Fig. 23. Rechallenge study with MC38 tumor cells. LID+US+αPDL1 treated animals were rechallenged by MC38 cells on day 40 and age-matched untreated mice were used as the control group. Shown are the individual tumor growth curves post rechallenge. CR=complete regression. (n = 5 mice for untreated and n = 7 mice for LID+US+αPDL1). Source data are provided as a Source Data file.

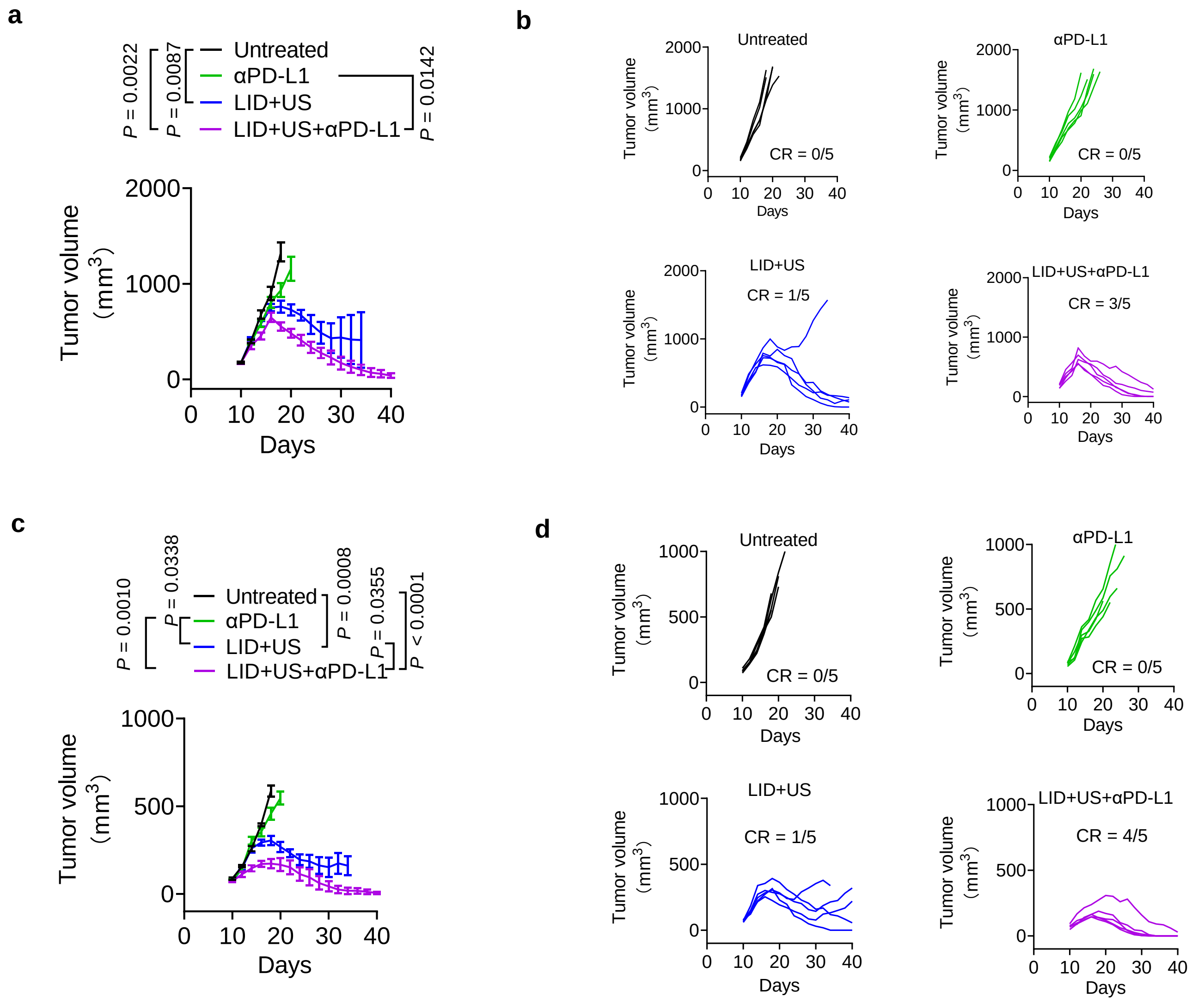

Supplementary Fig. 24. Therapeutic study on bilateral CT26 tumors. Balb/c mice were subcutaneously injected with 200,000 CT26 cells on the right flank and 100,000 CT26 cells on the left flank on day 0. On days 10, 13, and 16, tumor-bearing mice were i.v. injected with LID (DOX 0.5 mg/kg, ICG 4 mg/kg) or control formulations. On days 11, 14, and 17, ultrasound（2 W/cm^2, 50%, 1 MHz, 5 min）was performed for selected groups. On days 10, 13, and 16, the PD-L1 antibody (75 μg/dose) was i.p. injected for indicated groups. (**a-b**) The average and individual tumor growth curves for primary tumors (exposed to ultrasound) (n = 5 mice per group). (**c-d**) The average and individual tumor growth curves for distant tumors (not exposed to ultrasound). CR = complete regression. (n = 5 mice per group). Data were analyzed by two-way ANOVA with Tukey's multiple comparisons post test. Data represent mean ± SEM (**a,c**). Source data are provided as a Source Data file.

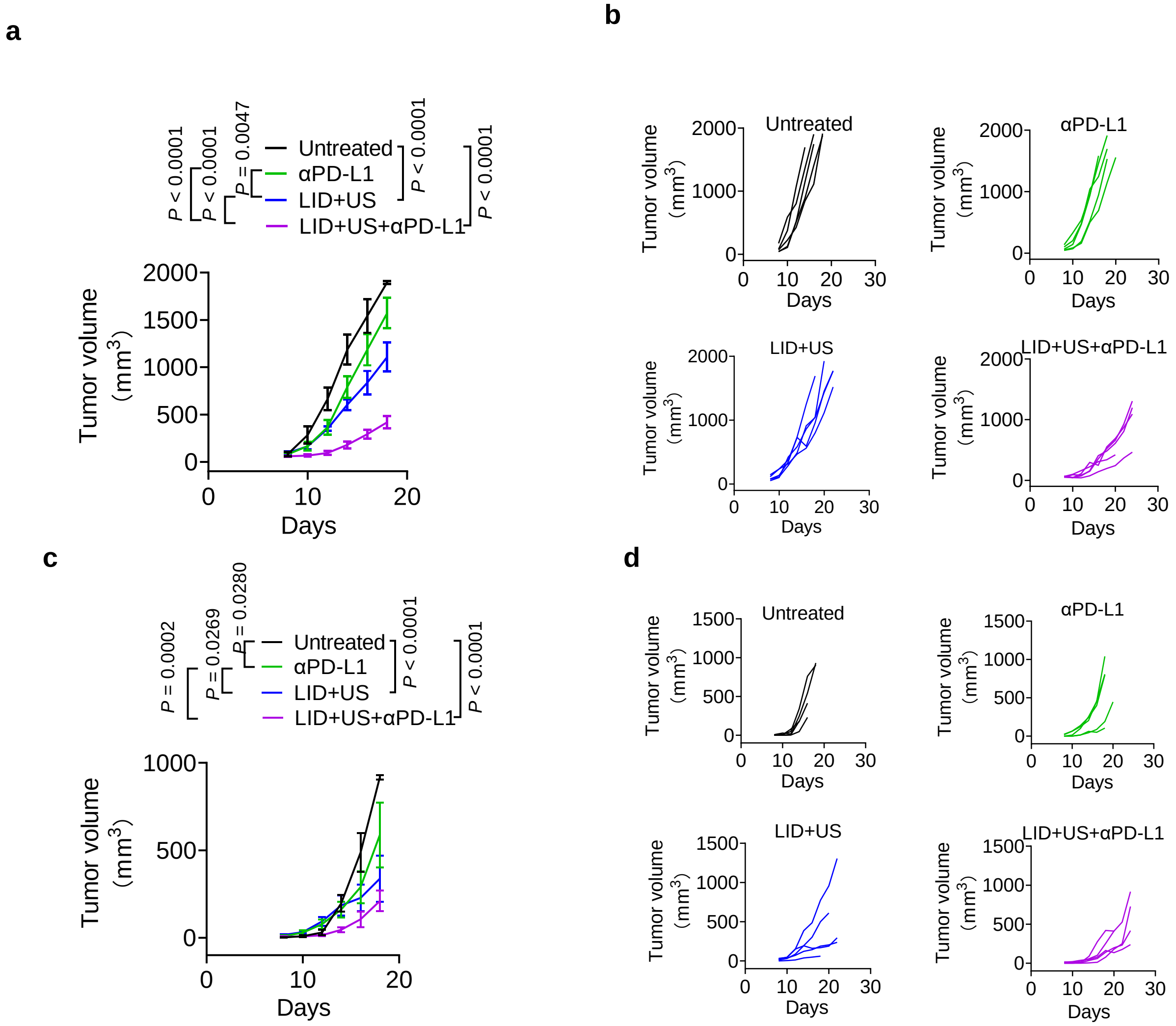

Supplementary Fig. 25. Therapeutic study on bilateral B16F10 tumors. C57BL/6 mice were subcutaneously injected with 200,000 B16F10 tumor cells on the right flank on day 0 and 100,000 B16F10 cells on the left flank on day 3. On days 8, 11, and 14, tumor-bearing mice were i.v. injected with LID (DOX 0.5 mg/kg, ICG 4 mg/kg) or control formulations. On days 9, 12, and 15, ultrasound（2 W/cm2, 50%, 1 MHz, 5 min）was performed for selected groups. On days 8, 11, 14, and 17 the PD-L1 antibody (75 μg/dose) was i.p. injected for indicated groups. (**a-b**) The average and individual tumor growth curves for primary tumors (exposed to ultrasound) (n = 5 mice per group). (**c-d**) The average and individual tumor growth curves for distant tumors (not exposed to ultrasound). (n = 5 mice per group). Data were analyzed by two-way ANOVA with Tukey's multiple comparisons post test. Data represent mean ± SEM (**a,c**). Source data are provided as a Source Data file.
