# Supplementary material for: Ultrasound-responsive low-dose doxorubicin liposomes trigger mitochondrial DNA release and activate cGAS-STING-mediated antitumour immunity
Source: Nat Commun. 2023 Jun 30;14:3877. doi: 10.1038/s41467-023-39607-x (PMC10313815; doi:10.1038/s41467-023-39607-x)
Supplement: Supplementary file 2 — Reporting Summary [file 41467_2023_39607_MOESM2_ESM.pdf]

nature portfolio

Corresponding author(s): Rui Kuai

Last updated by author(s): 2023-5-30

Reporting Summary

Nature Portfolio wishes to improve the reproducibility of the work that we publish. This form provides structure for consistency and transparency in reporting. For further information on Nature Portfolio policies, see our Editorial Policies and the Editorial Policy Checklist.

Statistics

For all statistical analyses, confirm that the following items are present in the figure legend, table legend, main text, or Methods section.

n/a	Confirmed	
☐	☒	The exact sample size (*n*) for each experimental group/condition, given as a discrete number and unit of measurement
☐	☒	A statement on whether measurements were taken from distinct samples or whether the same sample was measured repeatedly
☐	☒	The statistical test(s) used AND whether they are one- or two-sided<br>*Only common tests should be described solely by name; describe more complex techniques in the Methods section.*
☐	☒	A description of all covariates tested
☐	☒	A description of any assumptions or corrections, such as tests of normality and adjustment for multiple comparisons
☐	☒	A full description of the statistical parameters including central tendency (e.g. means) or other basic estimates (e.g. regression coefficient) AND variation (e.g. standard deviation) or associated estimates of uncertainty (e.g. confidence intervals)
☐	☒	For null hypothesis testing, the test statistic (e.g. F, t, r) with confidence intervals, effect sizes, degrees of freedom and P value noted<br>*Give P values as exact values whenever suitable.*
☒	☐	For Bayesian analysis, information on the choice of priors and Markov chain Monte Carlo settings
☐	☒	For hierarchical and complex designs, identification of the appropriate level for tests and full reporting of outcomes
☒	☐	Estimates of effect sizes (e.g. Cohen's d, Pearson's r), indicating how they were calculated

Our web collection on statistics for biologists contains articles on many of the points above.

Software and code

Policy information about availability of computer code

Data collection	Flow cytometric data were collected using BD FACSDiva Software v8.0. In vivo images were acquired using IVIS Lumina Living Image Software (v.4.5.5). DLS analysis was collected in Zetasizer software version 7.13.
Data analysis	Flow cytometry analysis was done in BD FACSDiva Software v8.0 and FlowJo (v.10.5) (Tree Star). In vivo images were analyzed using IVIS Lumina Living Image (v.4.5.5) Software. Statistical analysis was done in GraphPad Prism 8.0. DLS analysis was done in Zetasizer software version 7.13.

For manuscripts utilizing custom algorithms or software that are central to the research but not yet described in published literature, software must be made available to editors and reviewers. We strongly encourage code deposition in a community repository (e.g. GitHub). See the Nature Portfolio guidelines for submitting code & software for further information.

Data

Policy information about availability of data

All manuscripts must include a data availability statement. This statement should provide the following information, where applicable:

- Accession codes, unique identifiers, or web links for publicly available datasets
- A description of any restrictions on data availability
- For clinical datasets or third party data, please ensure that the statement adheres to our policy

The authors declare that data supporting the findings of this study are available within the article, Supplementary, or Source data files. RNA-sequencing datasets

have been deposited to NCBI-Sequence Read Archive (SRA) under accession code SRP439937. The link of this project is https://www.ncbi.nlm.nih.gov/bioproject/PRJNA977031. Source data are provided with this paper.

Research involving human participants, their data, or biological material

Policy information about studies with human participants or human data. See also policy information about sex, gender (identity/presentation), and sexual orientation and race, ethnicity and racism.

Reporting on sex and gender	*Use the terms sex (biological attribute) and gender (shaped by social and cultural circumstances) carefully in order to avoid confusing both terms. Indicate if findings apply to only one sex or gender; describe whether sex and gender were considered in study design; whether sex and/or gender was determined based on self-reporting or assigned and methods used. Provide in the source data disaggregated sex and gender data, where this information has been collected, and if consent has been obtained for sharing of individual-level data; provide overall numbers in this Reporting Summary. Please state if this information has not been collected. Report sex- and gender-based analyses where performed, justify reasons for lack of sex- and gender-based analysis.*
Reporting on race, ethnicity, or other socially relevant groupings	*Please specify the socially constructed or socially relevant categorization variable(s) used in your manuscript and explain why they were used. Please note that such variables should not be used as proxies for other socially constructed/relevant variables (for example, race or ethnicity should not be used as a proxy for socioeconomic status). Provide clear definitions of the relevant terms used, how they were provided (by the participants/respondents, the researchers, or third parties), and the method(s) used to classify people into the different categories (e.g. self-report, census or administrative data, social media data, etc.) Please provide details about how you controlled for confounding variables in your analyses.*
Population characteristics	*Describe the covariate-relevant population characteristics of the human research participants (e.g. age, genotypic information, past and current diagnosis and treatment categories). If you filled out the behavioural & social sciences study design questions and have nothing to add here, write "See above."*
Recruitment	*Describe how participants were recruited. Outline any potential self-selection bias or other biases that may be present and how these are likely to impact results.*
Ethics oversight	*Identify the organization(s) that approved the study protocol.*

Note that full information on the approval of the study protocol must also be provided in the manuscript.

Field-specific reporting

Please select the one below that is the best fit for your research. If you are not sure, read the appropriate sections before making your selection.

☒ Life sciences ☐ Behavioural & social sciences ☐ Ecological, evolutionary & environmental sciences

For a reference copy of the document with all sections, see nature.com/documents/nr-reporting-summary-flat.pdf

Life sciences study design

All studies must disclose on these points even when the disclosure is negative.

Sample size	Sample sizes were chosen based on our preliminary data from at least two pilot experiments and previously published results in the literature (doi.org/10.1038/s41565-018-0342-5, doi.org/10.1038/nmat4822).
Data exclusions	No data were excluded.
Replication	All experiments were repeated at least twice with similar results
Randomization	Mice were assigned randomly to experimental groups.
Blinding	The investigators were not blinded to allocation during experiments and outcome assessment since our data analyses are based on objectively measurable data.

Reporting for specific materials, systems and methods

We require information from authors about some types of materials, experimental systems and methods used in many studies. Here, indicate whether each material, system or method listed is relevant to your study. If you are not sure if a list item applies to your research, read the appropriate section before selecting a response.

Materials & experimental systems

n/a	Involved in the study
☐	☒ Antibodies
☐	☒ Eukaryotic cell lines
☒	☐ Palaeontology and archaeology
☐	☒ Animals and other organisms
☒	☐ Clinical data
☒	☐ Dual use research of concern
☒	☐ Plants

Methods

n/a	Involved in the study
☒	☐ ChIP-seq
☐	☒ Flow cytometry
☒	☐ MRI-based neuroimaging

Antibodies

Antibodies used

1. APC-anti-mouse CD8a Antibody (Clone: 53.6.7, Biolegend)
dilution 1:100
2. APC-anti-Mouse SIINFEKL/H-2Kb Purified eBio25-D1.16 (Clone: 25-D1.16, Invitrogen, eBioscience)
dilution 1:100
3. Purified anti-mouse CD16/32 Antibody (Clone: 93, Biolegend)
dilution 1:20
4. FITC-anti-Mouse CD80 (B7-1) Antibody (Clone: 16-10A1, Biolegend)
dilution 1:100
5. PE-anti-mouse CD11c Antibody (Clone: N418, Biolegend)
dilution 1:100
6. PE/Cyanine7-anti-mouse CD86 Antibody (Clone: GL-1, Biolegend)
dilution 1:100
7. APC/Cyanine7-anti-mouse CD4 Antibody (Clone: GK1.5, Biolegend)
dilution 1:100
8. FITC-anti-mouse CD3 Antibody (Clone: 17A2, Biolegend)
dilution 1:100
9. Alexa Fluor 647-8-OHdG Antibody (Clone: E-8, Santa cruz)
dilution 1:50
10. TBK1/NAK (D1B4) (3504T, Cell Signaling Technology)
dilution 1:1000
11. phosphor-TBK1/NAK (Ser172) (5483T, Cell Signaling Technology)
dilution 1:1000
12. IRF-3 (D83B9) (4302S, Cell Signaling Technology)
dilution 1:1000
13. phosphor-IRF-3 (S396) (4947S, Cell Signaling Technology)
dilution 1:1000
14. β-Actin (D6A8) Rabbit mAb (8457T, Cell Signaling Technology)
dilution 1:1000
15. Anti-Rabbit IgG (whole molecule)–Peroxidase antibody produced in goat (A9169, Sigma-Aldrich)
dilution 1:1000
16. STING/TMEM173 (A3575, Abclonal)
dilution 1:1000
17. anti-Mouse CD8α (BE0061, Bioxcell)
dilution 200ug/0.2mL
18. anti-Mouse PD-L1(BE0101, Bioxcell)
dilution 75ug/0.2mL
19. Calregulin (F-4) Alexa Fluor® 488 (sc-373863 AF488, Santa cruz)
dilution 1:100
20. T-select H-2Kb OVA Tetramer-SIINFEKL-PE (TS-5001-1C, MBL).
dilution 1:40

Validation

Antibody validation was provided by manufacture's website (cell images) and/or data provided in the manuscript.
1. APC-anti-mouse CD8a Antibody (Clone: 53.6.7, Biolegend)
https://www.biolegend.com/en-us/products/apc-anti-mouse-cd8a-antibody-150
2. APC-anti-Mouse SIINFEKL/H-2Kb Purified eBio25-D1.16 (Clone: 25-D1.16, Invitrogen, eBioscience)
https://www.biolegend.com/en-us/products/apc-anti-mouse-h-2kb-bound-to-siinfekl-antibody-7882
3. Purified anti-mouse CD16/32 Antibody (Clone: 93, Biolegend)
https://www.biolegend.com/en-us/products/purified-anti-mouse-cd16-32-antibody-190
4. FITC-anti-Mouse CD80 (B7-1) Antibody (Clone: 16-10A1, Biolegend)
https://www.biolegend.com/en-us/products/fitc-anti-mouse-cd80-antibody-41
5. PE-anti-mouse CD11c Antibody (Clone: N418, Biolegend)
https://www.biolegend.com/en-us/products/pe-dazzle-594-anti-mouse-cd11c-antibody-9846
6. PE/Cyanine7-anti-mouse CD86 Antibody (Clone: GL-1, Biolegend)

https://www.biolegend.com/en-us/products/pe-cyanine7-anti-mouse-cd86-antibody-3046
7. APC/Cyanine7-anti-mouse CD4 Antibody (Clone: GK1.5, Biolegend)
https://www.biolegend.com/en-us/products/apc-cyanine7-anti-mouse-cd4-antibody-1964
8. FITC-anti-mouse CD3 Antibody (Clone: 17A2, Biolegend)
https://www.biolegend.com/en-us/products/fitc-anti-mouse-cd3-antibody-45
9. Alexa Fluor 647-8-OHdG Antibody (Clone: E-8, Santa cruz)
https://www.scbt.com/zh/p/8-ohdg-antibody-e-8?requestFrom=search
10. TBK1/NAK (D1B4) (3504T, Cell Signaling Technology)
https://www.cellsignal.com/products/primary-antibodies/3504/
11. phosphor-TBK1/NAK (Ser172) (5483T, Cell Signaling Technology)
https://www.cellsignal.com/products/primary-antibodies/phospho-tbk1-nak-ser172-d52c2-xp-rabbit-mab/5483
12. IRF-3 (D83B9) (4302S, Cell Signaling Technology)
https://www.cellsignal.com/products/primary-antibodies/irf-3-d83b9-rabbit-mab/4302?site-search-type=Products&N=4294956287&Ntt=4302s&fromPage=plp&_requestid=1455950
13. phosphor-IRF-3 (S396) (4947S, Cell Signaling Technology)
https://www.cellsignal.com/products/primary-antibodies/phospho-irf-3-ser396-4d4g-rabbit-mab/4947?site-search-type=Products&N=4294956287&Ntt=4947s&fromPage=plp&_requestid=1458541
14. β-Actin (D6A8) Rabbit mAb (8457T, Cell Signaling Technology)
https://www.cellsignal.com/products/primary-antibodies/b-actin-d6a8-rabbit-mab/8457?site-search-type=Products&N=4294956287&Ntt=8457t&fromPage=plp&_requestid=1458604
15. Anti-Rabbit IgG (whole molecule)–Peroxidase antibody produced in goat (A9169, Sigma-Aldrich)
https://www.sigmaaldrich.cn/CN/zh/product/sigma/a0545
16. STING/TMEM173 (A3575, Abclonal)
https://abclonal.com.cn/catalog/A3575
17. anti-Mouse CD8α (BE0061, Bioxcell)
https://bioxcell.com/invivomab-anti-mouse-cd8a-be0061
18. anti-Mouse PD-L1(BE0101, Bioxcell)
https://bioxcell.com/invivomab-anti-mouse-pd-l1-b7-h1-be0101
19. Calregulin (F-4) Alexa Fluor® 488 (sc-373863 AF488, Santa cruz)
https://www.scbt.com/p/calregulin-antibody-f-4?requestFrom=search
20. T-select H-2Kb OVA Tetramer-SIINFEKL-PE (TS-5001-1C, MBL).
http://www.mbl-chinawide.cn/search012?keyword=TS-5001-1C

Eukaryotic cell lines

Policy information about cell lines and Sex and Gender in Research

Cell line source(s)	RAW-luciaTM ISG cells were purchased from InvivoGen. CT26, 4T1, and Hela cells were purchased from ATCC. MC38, HCT116, MDA-MB-231, and B16F10 cells were purchased from Cell Resource Center, IBMS, CAMS/PUMC. MC38-OVA cells were kindly provided by Dr. H. Peng (University of Chinese Academy of Sciences, Beijing). 4T1-luc cells were kindly provided by Dr. W. Liang (University of Chinese Academy of Sciences, Beijing).
Authentication	These cell lines were morphologically confirmed.
Mycoplasma contamination	No mycoplasma contamination was found.
Commonly misidentified lines (See ICLAC register)	No commonly misidentified cell lines were used.

Animals and other research organisms

Policy information about studies involving animals; ARRIVE guidelines recommended for reporting animal research, and Sex and Gender in Research

Laboratory animals	For in vivo studies, 6-8-week-old female BALB/c mice (Vital river, 18 g-20 g), 6-8-week-old female C57-BL/6 mice (Vital river, 18 g-20 g), and 6-8-week-old STING knockout female C57-BL/6 mice (GemPharmatech, 18 g-20 g) were used and housed in 12 light/12 dark cycle, 65-75°F (~18-23°C), and 40-60% humidity condition.
Wild animals	No wild animals were used.
Reporting on sex	The experiment was designed without considering the sex of the mice, and female mice were selected to ensure gender uniformity.
Field-collected samples	No field-collected samples were used.
Ethics oversight	All work performed on animals was in accordance with and approved by the Institutional Animal Care & Use Committee (IACUC) at Tsinghua University, Beijing.

Note that full information on the approval of the study protocol must also be provided in the manuscript.

Flow Cytometry

Plots

Confirm that:

☒ The axis labels state the marker and fluorochrome used (e.g. CD4-FITC).

☒ The axis scales are clearly visible. Include numbers along axes only for bottom left plot of group (a 'group' is an analysis of identical markers).

☒ All plots are contour plots with outliers or pseudocolor plots.

☒ A numerical value for number of cells or percentage (with statistics) is provided.

Methodology

Sample preparation	The sample preparation was described in the Methods.
Instrument	BD LSRFortessa SORP
Software	BD FACSDiva Software v8.0 was used for collection. BD FACSDiva Software and FlowJo were used for analysis.
Cell population abundance	Data on the abundance of relevant cell populations are provided in the manuscript.
Gating strategy	Cells were gated first by morphology to exclude cell debris, doublets were then gated out by FSC-A/FSC-H, SSC-A/SSC-H followed by exclusion of dead cells by gating on dye negative cells.

☒ Tick this box to confirm that a figure exemplifying the gating strategy is provided in the Supplementary Information.
