# Supplementary material for: Ultrasound-responsive low-dose doxorubicin liposomes trigger mitochondrial DNA release and activate cGAS-STING-mediated antitumour immunity
Source: Nat Commun. 2023 Jun 30;14:3877. doi: 10.1038/s41467-023-39607-x (PMC10313815; doi:10.1038/s41467-023-39607-x)
Supplement: Supplementary file 3 — Description of Additional Supplementary Files [file 41467_2023_39607_MOESM3_ESM.pdf]

Title: Supplementary Movie 1

Description: Cryo-ET of LID.
